# Importance of Type I and III Interferons at Respiratory and Intestinal Barrier Surfaces

**DOI:** 10.3389/fimmu.2020.608645

**Published:** 2020-12-11

**Authors:** Megan L. Stanifer, Cuncai Guo, Patricio Doldan, Steeve Boulant

**Affiliations:** ^1^ Department of Infectious Diseases, Molecular Virology, Heidelberg University Hospital, Heidelberg, Germany; ^2^ Department of Infectious Diseases, Virology, Heidelberg University Hospital, Heidelberg, Germany; ^3^ Research Group “Cellular polarity and viral infection”, German Cancer Research Center (DKFZ), Heidelberg, Germany

**Keywords:** interferons, type I interferon, type III interferon, interferon lambda, mucosal immunity, epithelial cells, respiratory epithelia, intestinal epithelium

## Abstract

Interferons (IFNs) constitute the first line of defense against microbial infections particularly against viruses. They provide antiviral properties to cells by inducing the expression of hundreds of genes known as interferon-stimulated genes (ISGs). The two most important IFNs that can be produced by virtually all cells in the body during intrinsic innate immune response belong to two distinct families: the type I and type III IFNs. The type I IFN receptor is ubiquitously expressed whereas the type III IFN receptor’s expression is limited to epithelial cells and a subset of immune cells. While originally considered to be redundant, type III IFNs have now been shown to play a unique role in protecting mucosal surfaces against pathogen challenges. The mucosal specific functions of type III IFN do not solely rely on the restricted epithelial expression of its receptor but also on the distinct means by which type III IFN mediates its anti-pathogen functions compared to the type I IFN. In this review we first provide a general overview on IFNs and present the similarities and differences in the signal transduction pathways leading to the expression of either type I or type III IFNs. By highlighting the current state-of-knowledge of the two archetypical mucosal surfaces (*e.g.* the respiratory and intestinal epitheliums), we present the differences in the signaling cascades used by type I and type III IFNs to uniquely induce the expression of ISGs. We then discuss in detail the role of each IFN in controlling pathogen infections in intestinal and respiratory epithelial cells. Finally, we provide our perspective on novel concepts in the field of IFN (stochasticity, response heterogeneity, cellular polarization/differentiation and tissue microenvironment) that we believe have implications in driving the differences between type I and III IFNs and could explain the preferences for type III IFNs at mucosal surfaces.

## Interferons and their Receptors

### Type I Interferons

Interferons (IFNs) were first discovered to interfere with the replication of influenza virus sixty years ago by Isaacs and Lindenmann (1). Since their discovery, many studies in humans and animals have started to unravel the molecular details of how IFNs elicit an intrinsic antiviral program in cells to control viral replication and spread (2, 3). IFNs form a diverse family of cytokines composed of three types designated as type I, II, and III IFNs. In humans and mice, type I IFNs are the largest family consisting of multiple subtypes of IFN-α (13 in humans, 14 in mice), as well as IFN-β, IFN-ϵ, IFN-κ, IFN-ω (humans) and IFN-ζ (mice) (4, 5). Type I IFNs have a broad range of functions including anti-pathogen activities (antiviral, antibacterial and antifungal), anti-proliferative functions and the ability to modulate innate and adaptive immunity (6, 7). While type I IFNs are ubiquitously expressed, there is evidence of cell type specific expressions of some IFN-α subtypes (8, 9). Type I IFNs are sensed by cells through the binding of the heterodimeric receptor composed of the IFN-α receptor 1 (IFNAR1) and the IFN-α receptor 2 (IFNAR2), which are expressed on all nucleated cells (10). All 17 type I IFNs are capable of binding the receptor complex but they do so with different affinities (11).

### Type II Interferons

The type II IFN family only has one member: IFN-γ. IFN-γ is produced predominantly by natural killer (NK) cells, natural killer T cells (NKT) and innate lymphoid cells (ILCs) and has been shown to be important for innate and adaptive immune responses (12). Additionally, it has been shown to play a key role in autoimmune and autoinflammatory diseases (13). IFN-γ binds to cells through the heterodimeric IFN- γ receptor 1 (IFNGR1) and the IFN-γ receptor 2 (IFNGR2) (14). Type II IFNs have been recently reviewed elsewhere (15) and this review will not focus on this cytokine.

### Type III Interferons

In 2003, two groups simultaneously discovered three new cytokines in humans that were able to block viral infection: IL29, IL28A and IL28B also known as IFN-λ1, IFN-λ2 and IFN-λ3, respectively (16, 17). As these cytokines exhibited similar functions as type I IFNs but were structurally unique they were designated as a new class of IFNs, the type III IFNs. In 2013, a new type III IFN (IFN-λ4) was identified (18). While the function of IFN-λ1, λ2 and λ3 in protecting and resolving pathogen infection is broadly accepted, the precise function of IFN-λ4 remains disputed. This controversy arises from the fact that exogenously produced IFN-λ4 shows antiviral activity, however whether cells can produce IFN-λ4 on their own remains debated (19). It is known that genetic polymorphisms (SNPs) in IFN-λ4 have been associated with the protein expression of IFN-λ4 which then impacts hepatitis C viral load, spontaneous clearance of the virus, and response to treatment (20, 21). Importantly, recent studies have shown that several human populations have lost the expression of IFN-λ4 suggesting that it has been deleterious for humans during the evolution process (22). Mice only express IFN-λ2 and IFN-λ3 as both IFN-λ1 and IFN-λ4 are pseudogenes. This review will not focus on IFN-λ4 but a comprehensive description of its biological activities has been recently reviewed (19).

Similar to type I IFNs, type III IFNs are expressed by most cell types in the body, however they are sensed by a more limited number of cells leading to cell type specific responses (23–25). Type III IFNs bind to a heterodimeric receptor composed of the type III IFN receptor (IFNLR1, also known as IL-28Rα) and the interleukin 10 receptor 2 (IL-10R2). The IL10R2 receptor is not only used by type III IFNs but is also used by other IL-10 family members such as IL-10, IL-22 and IL-26 (16, 17). While IL-10R2 is widely expressed in most cell types, IFNLR1 has a limited expression and is found in epithelial cells (*e.*g. intestine, lung, vaginal, and hepatocytes) (23–26) and some immune cells (DCs, pDCs, NK cells and neutrophils) (27–31).

## Production of IFNS

### Production of Type I and III IFNs

**Figure 1:**
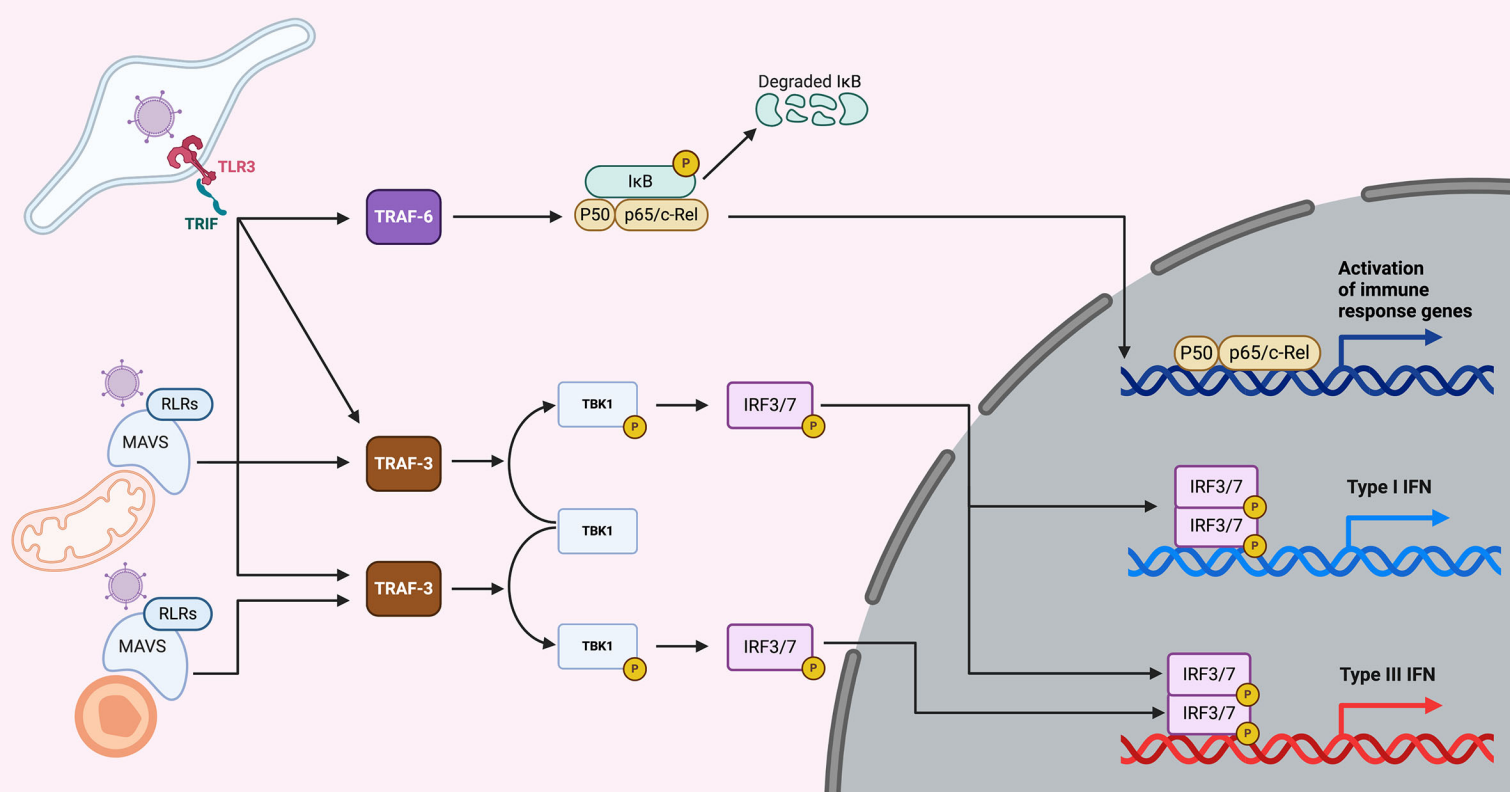
Overview of IFN production upon viral infection. Upon virus entry into cells, viruses are sensed by the TLRs (endosomes) or the RLRs (cytoplasm). TLR3 senses dsRNA (a main component of viruses or viral replication) and is located in endosomes. Upon sensing of dsRNA molecules, TLR3 and its adapter TRIF lead to the induction of both the NFкB and the IRF3/7 pathways, which lead to the induction of both pro-inflammatory cytokines and type I and III IFNs. Viral PAMPs located in the cytosol are recognized by RLRs and upon activation recruit the adapter protein MAVS. When MAVS is recruited to mitochondria, NFкB and IRF3/7 are activated leading to the induction of pro-inflammatory cytokines and type I and III IFNs. However, when MAVS is recruited to peroxisomes the signaling cascade leads to the induction of type III IFNs only.

Interferons are produced upon sensing of pathogen associated molecular patterns (PAMPs) by cellular pattern recognition receptors (PRRs). In a simplified view, as this is not the focus of this review, nucleic acids from the viral genome and intermediate products from virus replication are the main PAMPs for viruses. They are recognized by the Toll-like receptors (TLRs) and the RIG-like receptors (RLRs) (**Figure 1**). Activated PRRs recruit adapter proteins, such as Myeloid differentiation primary response 88 (MyD88), TIR-domain-containing adapter-inducing interferon-β (TRIF), and mitochondrial antiviral-signaling protein (MAVS) (32). The adapter proteins activate a series of downstream proteins and transcriptional factors, like interferon regulatory 3/7 (IRF3/7) and NFкB. Activated IRF3/7 undergo dimerizations and translocate to the nucleus, where they bind to enhancer/promoters of IFN genes, subsequently inducing the production and secretion of both type I and III IFNs (**Figure 1**) (33). Further details on the molecular mechanism used by cells to sense PAMPs and produce IFNs can be found in recent reviews (34, 35).

### Compartmentalization of PRRs for Production of Interferons

Pathogens can be sensed in different intracellular compartments depending on their mode of entry. In the case of viruses they can either infect the host by directly penetrating the plasma membrane or by being endocytosed and trafficking into the endosome compartment where they will be released into the cytosol (36). The site of entry will influence and dictate which PRR is most important for sensing viruses (TLR vs RLR) and as such will compartmentalize signal transduction leading to immune response and this could influence what kind of interferon is produced. This concept of compartmentalization of PRRs and downstream signaling has been pioneered through work on TLR4, which recognizes the bacterial component lipopolysaccharide (LPS). When TLR4 is located at the plasma membrane, stimulation of the receptor led to induction of pro-inflammatory cytokines through the MyD88 adaptor however, when TLR4 is internalized into the endosomes it leads to the production of interferons by recruiting the adaptor TRIF (37). The RLR adaptor MAVS is located at both the peroxisomes and the mitochondria (**Figure 1**) (38). Studies have shown that the peroxisomal MAVS leads to the production of type III IFN only, while mitochondrial MAVS can produce both type I and III IFNs (**Figure 1**) (39), however recent studies have contradicted this view and suggested that both type I and III IFNs can be produced from peroxisomal MAVS (40). Similarly, TLR3 was shown to be localized on the basolateral side of polarized human intestinal epithelial cells (41). This polarized localization of TLR3 led to a higher induction of interferons when cells were infected basolaterally with TLR3 activating viruses or stimulated with TLR3 agonist as compared to the apical side (41). This compartmentalization of TLR3 is key for intestinal epithelial cells which are in constant contact with the commensal flora. Having PRRs polarized to the basolateral side allows intestinal cells to partially tolerate the presence of apical commensals while remaining highly responsive against enteric pathogens that have crossed the intestinal epithelial barrier. These pathogens are sensed by the basolateral PRRs and will lead to a potent type III IFN response. On the contrary, the apical microbes (commensals and pathogens) are poorly sensed because few PRRs are localized at the apical side of intestinal epithelial cells (41). Further studies are required to determine whether other PRRs and their adaptors can be compartmentalized leading to differences in the production of type I and III IFNs. Most importantly it is critical, while studying intrinsic immune response, to not only consider which PRR is involved in sensing a pathogen but to also consider from where, within a cell, it is signaling and to integrate this in a tissue-like environment to allow for proper intracellular distributions of PRRs.

### Heterogeneity of Intrinsic Immune Response: Not All Infected Cells Produce IFNs

The textbook view of how PRRs sense pathogens and lead to the production of IFNs (**Figure 1**) would suggest that all infected cells in a population are equal: All cells will respond to pathogen infection and produce IFNs. However, recent studies have shown that each cell within a homogeneous cell population can respond differently. Work by O’Neal et *al.* showed that only a fraction of murine fibroblasts infected with West Nile virus produced IFN mRNA regardless of the viral load (42). Similarly, cell-to-cell variability was shown to regulate the ability of mouse fibroblasts infected with Sendai virus to produce IFN-β1 mRNA (43). Further studies have confirmed these observations and have shown that this heterogeneity is of cellular origin and not viral, and is due to intrinsic differences related to the activation and nuclear translocation of IFN regulatory transcription factors NFкB and IRF7 (44, 45). If IRF7 was not translocated into the nucleus then IFN-β1 was not made (44). These studies highlight that the ability of a virus to replicate and spread or be controlled by host defenses can be directly linked to the proportion of cells in a population that produce and respond to IFN (45). Whether there is a similar heterogeneity in the production of type III IFN is unknown as no studies have directly addressed this question. It is legitimate to speculate that a similar heterogeneity would exist for type III IFN because of the high similarity in the signal transduction pathways which lead to IFN production. However, it will be interesting to address if the cells act in pairs and those that do not produce type I IFN also do not produce type III or if the production of the two IFNs will be regulated independently.

## IFN-Mediated Signaling and ISG Production

### The Importance of JAKs in Interferon Signaling

**Figure 2:**
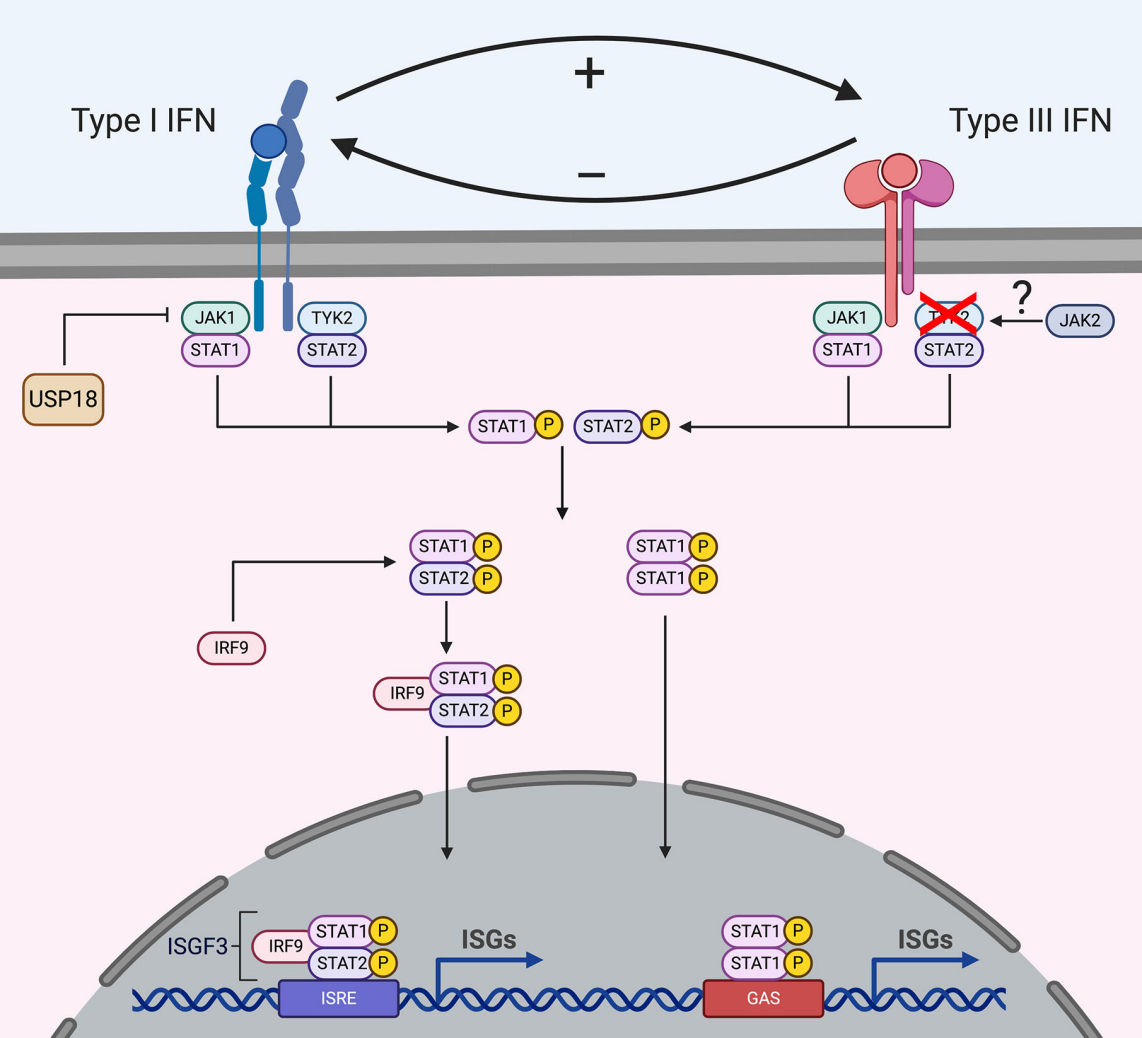
Signal transduction downstream type I and type III IFN receptors. Upon binding to their receptors, IFNs induce the activation of the JAK/STAT signaling cascade. Both type I and III IFNs use JAK1 for their signaling, while type I IFNs also require TYK2 activation, type III IFNs signal independently of TYK2. Several studies suggest that type III IFNs use JAK2 for their signaling while type I interferons do not require JAK2. However, how JAK2 interacts with the receptor complex is currently unknown. Following JAK activation, STATs are recruited and activated which leads to their dimerization and binding to IRF9 forming the ISGF3 complex or homodimer complexes which translocates into the nucleus and drives ISG production. Some ISGs act as negative regulators and the ISG USP18 is known to regulate type I IFN signaling but not type III IFN signaling.

Following production and secretion of type I and III IFNs, these two cytokines will bind to their specific receptors in an autocrine (the secreting cells) and paracrine manner (the bystander cells) to activate complex signal transduction pathways which will induce transcriptional responses that will ultimately result in the development of an antiviral state in the stimulated cells (**Figure 2**). Both the type I and III IFNs induce the JAK/STAT signaling cascade leading to the induction of interferon stimulated genes (ISGs) (**Figure 2**) (26, 46–48). IFNs first bind one receptor chain with high affinity (IFNAR2 or IFNLR1), and then recruit the low-affinity chain (IFNAR1 or IL-10R2) to form a signaling-competent ternary complex (49–51). Upon binding, the extracellular part of the receptors induces the conformational change of the intracellular part of the receptor subunits, which causes receptor dimerization. Receptor dimerization activates receptor-associated Janus kinases (JAK), TYK2 and JAK1, which mediate the phosphorylation of tyrosine residues on the intracellular part of IFN receptors (52, 53). JAK1 is associated with IFNAR2 and IFNLR1 while TYK2 is associated with IFNAR1 and IL-10R2 (**Figure 2**) (54–58). JAK1 is critical for the activation and signaling of both type I and III IFNs. Importantly, JAK1 mutations have not been found in humans and are embryonic lethal in mice suggesting that they play a critical role in immune responses and development (59, 60).

Recent reports have uncovered that while TYK2 is required for type I IFN signaling, it is dispensable for type III IFN signaling (61, 62). Several mutations in the Tyk2 locus have been identified in patients, however they do not show a high susceptibility to viral infections (61, 63). As TYK2 appears dispensable for type III IFN signaling, it is likely that in these patients the absence of increased susceptibility to pathogens is the result of type III IFNs providing first line protection, at least at mucosal surfaces. This model is supported by recent results where TYK2 knock-out murine intestinal epithelial cells treated with type III IFNs maintain their ability to produce ISGs and protect against virus replication (unpublished). Additionally, TYK2 knock-out mice pretreated with type III IFN prior to influenza infection were protected against viral infection in respiratory epithelial cells, while type I IFN pretreatment did not confer protection (unpublished). How signaling downstream the type III IFN receptor is transduced in the absence of TYK2 is unknown, however, it is tempting to speculate that another kinase takes over the function of TYK2. Interestingly, cells depleted of JAK2 or cells treated with specific JAK2 inhibitors are able to respond to type I IFNs and not type III IFNs suggesting that JAK2 could act in place of TYK2 for type III IFN signaling (**Figure 2**) (30, 39, 64).

### STATs in Interferon Signaling

Following JAK activation and receptor phosphorylation, signal transducer and activator transcription (STAT) proteins are recruited to the complex. STATs are subsequently phosphorylated by JAKs and activated STATs form STAT1/2 heterodimers which bind to IRF9, forming the transcription factor interferon-stimulated gene factor 3 (ISGF3). ISGF3 transfers to the nucleus and binds interferon-stimulated response elements (ISREs), driving the transcription of interferon-stimulated genes (ISGs) (**Figure 2**). While STAT1/2 are the main proteins used in IFN signaling, other STATs are found to be activated and play cell type specific functions. Following type I IFN binding, STAT1–6 have all been shown to participate in the antiviral and anti-proliferative actions of these IFNs (65, 66). STAT1–3 are induced in all cell types, while STAT4–6 are cell type specific (67–69). However, which specific ISGs are produced upon STAT4–6 activation needs to be further investigated. Similar to type I IFNs, type III IFNs also induce STAT1–5 (**Figure 2**) (70, 71). However, to date, it remains unclear whether differences in the phosphorylation of the different STAT proteins are responsible for the differences in kinetics and magnitude of ISG expression observed between type I and III IFNs (See section “Interferon specific ISGs”).

### Negative Regulation of Interferon Signaling

Probably the most important step in mounting an antiviral response is the ability of cells to turn it off. Failure to arrest IFN signaling in tissues leads to inflammatory disorders in patients and interferonopathies (72). These disorders arise when cellular pathways fail to regulate IFN signaling and are often treated by blocking IFN signaling through the use of JAK inhibitors (73).

The suppressor of cytokine signaling (SOCS) (e.g. SOCS1 and SOCS3) are considered the most potent negative regulators used by cells to control type I IFN signaling as they can directly interact with TYK2 interfering with its activation (74). SOCS1 specifically acts by modulating the activity of IFNAR1 through downregulating TYK2 expression (75). Overexpression of SOCS1 in hepatic cells lines has been shown to also act on type III IFN leading to decreases in ISG production (76). Importantly, *in vivo* studies using SOCS1 knock-out mice showed increased ISG induction in the liver in response to type III IFNs while the lung and gut were only mildly affected (76). As lung and gut cells have been shown to be TYK2 independent, this suggests that either SOCS1 acts through another method to impact type III IFN signaling or that there are organ-specific differences in the regulation of IFNs.

JAK1 signaling can be regulated by the ISG ubiquitin-specific protease 18 (USP18). USP18 is induced upon both type I and type III IFN treatment, however it specifically regulates type I IFN signaling by binding to IFNAR2 (**Figure 2**) (77). Upon binding, USP18 acts as a negative regulator by preventing the interaction of JAK1 with IFNAR2 and thereby limiting type I IFN signaling. Interestingly, as type I IFNs bind to the receptor complex with different affinities, USP18 exerts its functions in a subtype dependent fashion with USP18 blocking IFN-α subtypes more than IFN-β1 (77–79). High USP18 levels are also suggested to be the reason that many hepatitis C infected patients show a refractory phenotype to IFN-α based antiviral therapy (80). Even though type III IFN signaling requires JAK1, it is not affected by USP18 as USP18 specifically targets and binds IFNAR2 and not IFNLR (81).

### Regulation of Antiviral Functions

Beside activating the JAK/STAT signaling pathway, both type I and type III IFNs also induce the mitogen-activated protein kinases (MAPKs). Interestingly, in human intestinal epithelium cells, both type I and III IFNs activate MAPK signaling pathways however only type III IFNs require them for their antiviral functions. Intestinal cells treated with MAPK inhibitors blocked the ability of type III IFNs to control virus infection while type I IFNs’ antiviral properties stayed intact (71). These observations suggest that to properly control viral infection, cells not only rely on ISGs made downstream JAK/STAT but that other parallel signaling pathways might be involved in determining the final outcome of infection by providing assistance to the main IFN-mediated antiviral signal. Whether this dependency on MAPK is intestinal cell specific and whether these differences participate in the regulation of ISG expression following type I and type III IFN stimulation of cells remains to be carefully addressed.

Interestingly, the signaling pathways downstream type I and III IFNs are interconnected and influence each other (82). Studies in human intestinal epithelial cells lacking either the IFNAR1 or IFNLR1 showed that the presence of a functional type III IFN receptor negatively regulates type I IFN signaling and antiviral activity, whereas the presence of type I IFN receptor positively reinforces type III IFN signaling and function (**Figure 2**) (82). These results suggest that studies which employ cells depleted of either IFN receptor, might show differences in responses to pathogens or signaling cascades that are not only due to the lack of the knocked-out receptor but also due to impaired signaling of the remaining receptor. Additionally, in tissues where one IFN receptor is naturally absent (*e.g.* murine intestinal cells which lack IFNAR, see section *Role of Type I and III IFNs in the Murine Intestine*), the properties of the remaining IFN receptor (*i.e.* IFNLR) could be weakened or enhanced.

### Interferon-Specific ISGs

Over the past 17 years many studies have compared the differences between the ISGs induced upon type I and III IFNs stimulation in several mucosal tissues (*e.g.* intestine, lung and liver). These studies have revealed that while there is a core set of ISGs (*e.g.* IFIT1, MX1, USP18) induced in all tissues evaluated, there are others that may be tissue-specific (e.g. RSAD2 and GIP3 are highly induced in hepatocytes upon IFN treatment but are not induced in intestinal cells, where intestinal cells highly upregulate CXCL10 and BST2 which are absent in hepatocytes) (46, 47, 83, 84). However, defining which ISGs are specific to type I or type III IFN and which ones are tissue specific is very challenging. The reason for the difficulty in drawing a conclusive picture of type I vs. type III IFN signaling is that each study has used different amounts of IFNs to induce ISG production. Most importantly, evaluation of the IFN-mediated response was performed at different times post-IFN stimulation and this could severely impact which ISG is detected.

One of the predominant differences between the type I and the type III IFN-mediated immune response is that both cytokines induce ISG expression with very different kinetics. Human intestinal epithelial cells treated with either IFN-β1 or IFN-λ1-3 were shown to induce a similar set of ISGs but these ISGs were induced with different magnitudes and at different times post-IFN stimulation (46). Type I IFN showed a fast and strong induction of many ISGs compared to type III IFNs which showed a delayed induction of ISGs with a lower magnitude (46). This temporal induction of ISGs leads to a unique antiviral environment created by each IFN. These differences in the magnitude and temporal expression of ISGs appears to not be tissue-specific but intrinsic to both IFNs, as similar differences in ISG expression kinetics were also seen in respiratory epithelial cells and liver cells which also showed higher and faster induction of type I IFNs compared to type III IFNs (47, 83, 85–87). Importantly this delayed induction of ISGs by type III IFNs was not due to lower receptor levels, as overexpressing IFNLR1 did not lead to a faster induction of ISGs suggesting that type I and III IFNs uniquely regulate their signaling cascades (46).

IFN specific ISGs have been uncovered for type I and III IFNs in respiratory epithelial cells, liver cells, and intestinal epithelial cells (84–86). Studies in respiratory epithelial cells and liver cells revealed that IRF1 is induced both at the RNA and protein level only upon type I IFN treatment, however when cells were co-treated with type I and III IFNs the expression of IRF1 is prolonged suggesting that type III IFNs stabilize its expression (85, 86). The lack of induction of IRF1 and its proinflammatory downstream targets by type III IFNs has been suggested to explain why type III IFNs limit tissue damage following viral infection (85). Interestingly, mouse intestinal cells were found to produce type III IFN specific ISGs (*i.e.* Mmp7, Serpinb1a, and Csprs) (84). These IFN-λ2 specific genes were only found in the intestine and were not induced in the lung or bone marrow derived dendritic cells (BMDCs) following IFN treatment further supporting the model that tissues have unique sets of ISGs (84).

Our current understanding of type I and III IFN-mediated signaling suggest that while the main signal transduction pathways are very similar between both IFNs, there are unique differences between each cytokine (**Table 1**) that may provide IFN-specific control of pathogen infections. Although over the years, the signal transduction pathways downstream of the type I IFN receptor have been highly studied, many gaps are remaining in our understanding of the signaling pathways induced by type III IFNs. A systematic side-by-side comparison would be necessary to fully appreciate the differences in signaling pathways and the molecular mechanisms leading to antiviral function activated upon type I and type III IFN-mediated responses.

**Table 1:** Similarities and differences of type I and III IFNs.

	Type I IFN	Type III IFN
IFN production	Produced downstream TLR3, TLR4 (endosomes), RLRs, STING (32–35) Produced by MAVS located on mitochondria (38, 39)	Produced downstream TLR3, TLR4 (endosomes), RLRs, STING (32–35) Produced by MAVS located on both mitochondria and peroxisomes (38, 39)
Receptor distribution	Receptor expressed by all cells in the body (10)	IFNLR receptor chain is only expressed in epithelial cells and in some immune cells (DCs, pDCs, NK cells and neutrophils) (23–31)
JAK/STAT signaling	Requires JAK1 (JAK2 independent) (30, 39, 55, 57–59, 64, 72) Signaling is TYK2 dependent (52–55)	Requires JAK1 and JAK2 (30, 39, 72) Signaling is TYK2 independent (61–63)
Other pathways	Negatively regulated by IFNLR (82)	Positively regulated by IFNAR (82) Requires MAPKs for its antiviral activity (71)
Magnitude and kinetics of ISG induction	High magnitude of ISG induction (46–48, 83, 85) Fast induction and fast decrease in ISG expression (46–48, 83, 85) 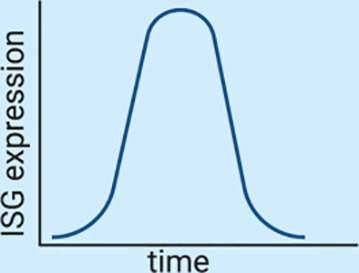	Low magnitude of ISG induction (46–48, 83, 85) Slow but sustained induction of ISGs (46–48, 83, 85) 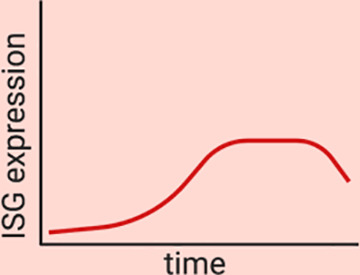
Negative regulators	USP18 downregulates IFN-mediated signaling (77, 79, 81) SOCS1 and 3 downregulate IFN-mediated signaling (74–76)	SOCS1 downregulates signaling is some tissues (76)
IFN-specific ISGS	IRF1 (85–87)	Mmp7, Serpinb1a, and Csprs (84)

### Heterogeneity in IFN Sensing: Not All Cells Respond to IFN

**Figure 3:**
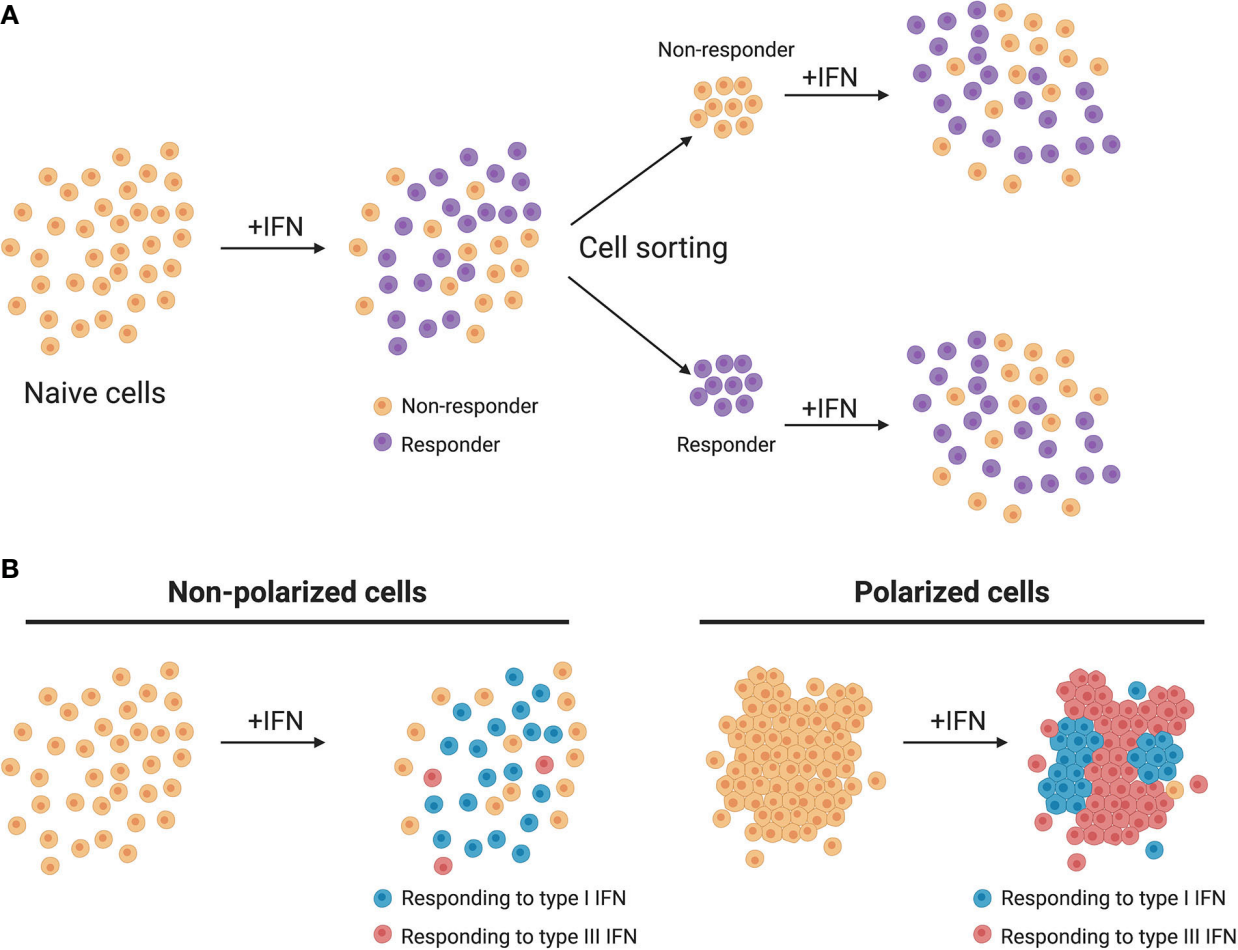
Heterogeneity of IFN production and response. **(A)** Homogeneous cell cultures treated with IFN (+IFN) respond in a heterogeneous manner. Upon sorting and restimulation with IFN, non-responding cells display a similar distribution of responding cells as the naive population. **(B)** In murine models, non-polarized cells respond mainly to type I IFNs while polarized cells respond mainly to type III IFNs.

Upon interferon treatment it is accepted that signal transduction leads to the nuclear translocation of the ISGF3 complex and subsequent activation of ISGs in all treated cells (**Figure 2**). Nevertheless, recent studies have revealed that although a cell culture population is genetically homogeneous, cells within it could still respond differently to external stimuli, thus producing distinctive amounts of mRNA (88). It was originally thought that ISGs are produced in a binary manner, meaning that the presence of IFN switches them from an “OFF” to an “ON” state (or *vice versa*) (89). However, in recent years several groups have shown that seemingly homogeneous cell culture systems respond to type I IFN treatment in a heterogeneous manner. Mouse fibroblasts treated with IFN-β1 and analyzed in a single cell manner showed that ISG induction was asynchronous and that the magnitude of ISG induction varied between cells (44). Importantly, a subpopulation of IFN treated fibroblasts never responded regardless of IFN concentration indicating that part of the population became refractive to IFN stimulation (**Figure 3A**) (44). A similar subpopulation of non-responding cells was also found in both human liver cells and human airway epithelial cells stimulated with IFN-α (45, 90). In both of these human cell lines, the non-IFN responding cells were not defective in IFN sensing as sorting of the non-responding cells and re-stimulating them with IFN-α induced activation of ISGs with a similar proportion of cells responding and non-responding to IFNs (**Figure 3A**) (45, 90). Mathematical models have shown that a higher initial level of the transcription factor IRF9 determines the intensity and speed with which cells are able to respond to IFNs, and thus, differences in the levels of the ISGF3 complex members could play a key role in the responsiveness to IFNs (91). It is important to consider that differences in the basal levels of many proteins involved in signal transduction downstream the IFN receptors are likely to give rise to different outcomes upon IFN stimulation.

This heterogeneity in ISG induction is not exclusively found in type I IFNs, as recent studies have shown that 90% of a clonal population of mouse derived IECs responded to IFN-β1, whereas 55% of these cells responded to type III IFNs (92). This discrepancy between the number of cells that responded to type I and type III IFNs implies that different mechanisms regulate whether a cell responds or not to either IFN. This may provide a unique opportunity for cells that are normally responsive to both IFNs (e.g lung and gut epithelial cells) to favor one IFN over the other to promote an IFN-specific signaling/function. This lower cellular responsiveness against type III IFNs was also seen in human IECs, where even at very high concentrations type III IFN was never able to fully protect all cells from virus infection (85-90% inhibition) while type I IFN was (46). Histone deacetylases (HDACs) were described to play a role in regulating the sensitivity of epithelial cells to IFN-λ, as pretreatment of mouse intestinal cells with HDAC inhibitors significantly increased the number of IFN-λ responding cells. It is possible that the sensitivity of cells to IFNs is directly or indirectly regulated at the epigenetic level, and that a lack of synchronicity in these regulatory pathways causes delays or insensitivity to either or both type I and III IFNs.

Furthermore, as type III IFNs act on epithelial surfaces it is important to consider their polarization state. Many experiments in laboratory settings use epithelial cells in sparse conditions whereas in the normal tissue environment they are tightly connected and polarized. The state of the cells is critical when evaluating responsiveness to IFNs as mouse intestinal cells have shown to be more sensitive to IFN-λ when reaching a polarized status (**Figure 3B**) (92) while human intestinal epithelial cells become less responsive to IFNs when polarized (71). Understanding the molecular mechanism of how within a population cell density, polarization status and epigenetic inheritance influence responsiveness to either IFN is a promising research axis that will help us to delineate the differences observed between different tissues and between different species.

## IFN Lambda in Mucosal Immunity

The main difference that places type I and type III IFN apart lies in the fact that the type III IFN receptor expression is restricted to a subset of cells (23), providing these cells a unique way of protecting themselves against pathogen challenges. Research has focused initially on evaluating how type I and III IFNs control pathogen infections in the intestinal tract, the respiratory tract, the liver, the blood brain barrier and more recently the female reproductive tract. In this review we focus on the intestinal and respiratory epithelial cells because there is increasing evidence that type I and III IFNs are critical for both the intestinal and airway epithelium not only by mediating the antiviral response but also by impacting/regulating the epithelium themselves and by controlling and maintaining adaptive immune responses and the integrity of the epithelial barrier. More details on the role of IFNs in the female reproductive tract and the blood-brain-barrier can be found in a recent review (93).

### Role of Type I and III IFNs in the Murine Intestine

The epithelial cells lining the intestinal tract play a unique role in regulating immune-homeostasis. These cells must be able to tolerate the huge commensal load present in the lumen of the gut and be responsive to invasive pathogens. In the intestinal tract, type III IFNs have been shown to play a key role in helping to maintain this balance and protecting the intestinal epithelial cells lining the gut from enteric pathogens while limiting excessive immune responses leading to tissue damage (24, 94–97). Upon enteric virus infection, murine IECs preferentially express type III IFNs over type I IFNs (95, 97). It has been shown that epithelial cells express higher levels of IFNLR1 and lower levels of IFNAR1 and IFNAR2 compared to the underlying lamina propria (95). This compartmentalization of the IFN receptors also favors IFN-λs as a first line defense against enteric pathogens (24). Using rotavirus as a model enteric virus, which predominantly infect epithelial cells, it was shown that mice lacking the type I IFN receptor were able to control rotavirus infection while mice lacking type III IFN receptor showed increases in virus replication, *de novo* virus production and damage to the intestinal epithelium (24, 97).

Type I IFNs are not dispensable for enteric infections. While they do not act to protect the epithelial surface, they play a key role in protecting against systemic spread of the viruses. Infection experiments using the enteric virus reovirus, which can also spread systemically following infection and replication in the GI tract, confirmed the critical role of type III IFN in protecting IECs against viral infection. Most importantly, it was shown that the type I IFN system was responsible for controlling the systemic dissemination of reovirus (95). Similar results were obtained using mouse norovirus (94).

The ultimate proof that type III IFN was the main player protecting IECs from enteric virus infection was provided by experiments performed in mice where the function of IFN-λ was only disrupted in IECs and by curing enteric infection using IFN-λs. Using mice with intestinal-specific conditional knock-out of the IFNLR1 it was shown that IFN-λ signaling in IECs is protective against enteric virus infections even in mice lacking an adaptive immune system (Rag-1^-/-^) and that depletion of IFN-λ signaling from IECs resulted in an increase in norovirus, rotavirus and reovirus replication and fecal shedding (98). Complementarily, it was shown that administration of IFN-λs in mice could resolve persistent norovirus infection also in the absence of adaptive immunity (94).

Interestingly, recent evidence suggests that this spatial functional compartmentalization of IFNs at the intestinal epithelium, where the type III IFN is important for IECs protection while type I IFN is set to prevent systemic spread, is not genetically encoded but acquired in older animals. Adult mice only use the type III IFN receptor to control rotavirus infection in the gut (24, 95). On the contrary, neonatal mice appear to require both type I and III IFN receptors to efficiently protect IECs against rotavirus infection (99). Complementarily, while murine IECs do not respond to type I IFN *in vivo*, it was shown that they become responsive when isolated and stimulated with type I IFN cytokine *ex vivo* (24, 92). This observation is consistent with the fact that mouse intestinal organoids are also responsive to both IFNs (92).

The molecular origin for this reversion of IECs toward responsiveness to type I IFN is unknown. It is possible that following isolation from the intestinal epithelium, even when grown as organoids, IECs partially dedifferentiate and lose regulatory mechanisms that normally dampen the type I IFN mediated response. Another possible explanation is the presence of the commensal flora in the lumen of the gut, which is absent at birth but grows in number and complexity with time. The presence of these commensals might interfere with the type I IFN system in IECs. A relationship between IFNs and the microbiota was previously described, where commensals seem to negatively regulate the type III IFN-mediated clearance of persistent enteric virus infection (100). Additionally, IECs depleted of IFNAR1 result in a significant change of the microbiota composition likely as a result of changing the number of Paneth and Goblet cells in the epithelium (101). This is an interesting observation as it suggests that although type I IFN is not important to protect IECs against viral infection, the type I IFN pathways might still be active to regulate other functions and help promote homeostasis of the intestinal epithelium.

### Role of Type I and III IFNs in the Human Intestine

Similar to murine cells, human intestinal epithelial cells respond to enteric virus infection by inducing a strong upregulation of type III IFNs transcripts while type I IFN transcripts are upregulated to a much lesser extent (41, 71, 102, 103). This leads to the preferential expression and secretion of type III IFNs, and thus to a protective effect of this cytokine on the surrounding epithelial cells expressing the type III IFN receptor (71). While the importance of type III IFN and dispensability of type I IFN in protecting IECs is well established in mice with the use of transgenic animals, it was only recently demonstrated that IFN-λs were keys to protect the human gut. It was shown that type III IFN controls SARS-CoV-2 infection of human intestinal epithelial cells. Human intestinal cells lacking the type I IFN receptor behaved similarly to wild type cells, whereas cells depleted of the type III IFN receptor showed increased virus infection, replication, and *de novo* virus production (96). All together this has led to the model in which the functions of type I and type III in the murine intestines are compartmentalized; type I IFNs protect the lamina propria and virus dissemination to the body and type III IFNs protect the epithelial surface itself (24, 95, 98).

### Role of Type I and III IFNs in Respiratory Epithelial Cells

Similar to IECs, production of IFN-λs upon viral infection is a characteristic response of lung epithelial cells (95, 104). Overall, lung epithelial cells appear to favor the production of IFN-λs compared to type I IFN upon influenza A virus (IAV) infection (29, 104). Many studies using mice lacking either the type I or type III IFN receptors could show that either IFN was able to control infections by influenza viruses, respiratory syncytial virus or human metapneumovirus, suggesting that type I and III IFNs played a redundant function in the lung (25, 104). While the epithelial cells are the most responsive cells to type III IFNs due to the specificity of IFNLR1 expression, major differences in their ability to control virus infections are observed when comparing the upper and lower respiratory tract. In infection models where high doses of IAVs are used to infect the lower respiratory tract, both type I and type III IFNs are important to combat infection (25, 29, 104). On the contrary, if lower doses of IAVs are used and/or administered in a more physiological manner *via* nasal infection, the critical role of type III IFN for controlling IAV becomes much more apparent (29, 105). A recent study using mice lacking the type I or III IFN receptors in either neutrophils or epithelial cells specifically showed that each IFN had a unique effect in controlling influenza infection (29). This study revealed that upon influenza infection of respiratory epithelial cells, type III IFNs were produced first, and if the influenza virus load stayed low they were the most important IFNs used to clear the infection. Upon a greater viral load, type I IFNs were required to control the infection (29).

### Role of Type I and III IFNs in Immune Cells at Mucosal Surfaces

An important growing concept in the field of type III IFNs, is that this cytokine is not only critical to control, clear and prevent pathogen infection at the level of the epithelium but it is also playing a role in providing long term immunity by stimulating adaptive immunity. Type III IFNs protect against long term infection and are required to reduce spreading of influenza virus to littermates (105) and are important for enhancing mucosal adaptive immunity by promoting antigen-dependent germinal center reactions in draining lymph nodes (106, 107). Additionally, a recent study has shown that mice lacking IFNLR1 showed impaired CD8^+^ T cell and antibody responses following infection by a live-attenuated influenza virus (106). Influenza infection induced the release of IFN-λ, which triggered M cells to produce thymic stromal lymphopoietin (TSLP) in the upper airways. The release of TSLP then stimulated migratory dendritic cells and boosted antigen-dependent germinal center reactions in draining lymph nodes (106). The IFN-λ-TSLP axis also promoted production of the immunoglobulins IgG1 and IgA only when applied intranasally, suggesting that it required mucosal surfaces for its action (106).

IFN-λ acts on neutrophils to not only control virus infections but also fungal infections of the respiratory tract, as was recently highlighted in studies evaluating *Aspergillus fumigatus* infection in mice. Mice lacking the IFNLR1 were unable to activate a neutrophil response and showed higher fungal loads, a more aggravated disease in the lungs and severe fungal invasion (108). While these studies have clearly shown that murine neutrophils respond to IFN-λ and use it to help in pathogen clearance, the ability of human neutrophils to be activated in response to type III IFNs remains controversial (30, 31, 109). Whether tissue specific immune cells also play a role in regulating type I and III IFN responses in the intestinal epithelium have not been addressed.

### Role of Type I and III IFNs in Maintaining Barrier Functions

Both type I and III IFNs have been shown to play a role in tightening barriers at mucosal interfaces. Following respiratory infection by *S. pneumoniae*, mice upregulated the IFN-β1 transcript, which was critical to control bacterial invasion (110). Mice lacking IFNAR1 or mice treated with a IFNAR neutralizing antibody showed an increase in bacteremia. Further studies showed that IFN-β1 induced the production of tight junction proteins and prevented transmigration of bacteria across the epithelial membrane (110). Similarly, type III IFNs were shown to protect human intestinal epithelial cells from *Salmonella enterica* serovar *Typhimurium* infection (111). Intestinal cells treated with both type I and III IFNs increased their barrier function and prevented the passage of dextran molecules through the epithelial membrane, however type III IFN was more efficient at blocking transmigration of *Salmonella* and the epithelial damage caused by *Salmonella* infection (111). Type I and III IFNs have also been shown to play a key role in tightening the blood-brain-barrier (BBB) which has been recently reviewed (93).

## Conclusion and Future Perspectives

The type III IFN system is no longer considered just a redundant system to type I IFNs but it is now fully recognized as providing a novel arsenal to the host to protect specific cells and tissues against pathogen challenges. The observation that type III IFNs can specifically provide efficient protection against pathogens in the gut and the lung (**Table 2**) (24, 95, 104), which could even be sterilizing in the absence of adaptive immunity, (94) has placed type III IFNs with a unique therapeutic potential. Within the current SARS-CoV-2 pandemic, it was quickly discussed that type III IFN could help to curtail viral replication while limiting the tissue damage that could be induced by type I IFN.

**Table 2:** Role of type I and III IFNs in respiratory and intestinal epithelial cells.

	Lung	Gut
Antiviral functions in epithelial cells	Type III IFNs control viral infection in the upper respiratory tract (25, 109) Both type I and III IFNs control viral infection in the lower respiratory tract (104) Type III IFN acts first and type I IFN acts when infections persist (29)	Type III IFNs act on epithelial cells to control infection (23–25, 71, 95, 96, 98, 99) Type I IFNs act on lamina propria to prevent systemic spread (23, 24, 95, 99) Type III IFNs can control virus infection in epithelial cells in the absence of adaptative immune response (94)
Organ-specific ISGs	Unknown if lung epithelial cells produce ISGs that are not induced in intestinal epithelial cells	Mmp7, Serpinb1a, and Csprs expressed in gut but not in lung following IFNλ2 treatment (84)
Importance of IFN signaling in immune cells	Type III IFNs are needed to reinforce adaptative immune responses (30, 106) Neutrophils depleted of IFNLR were unable to control fungal infections in the lung (108)	Innate lymphoid cells produce IL-22 which synergize with type III IFNs to induce higher levels of ISGs and increase antiviral (rotavirus) protection of murine IECs (97, 117)
Barrier functions of epithelium	Type I IFNs and IFNAR are required to maintain the lung epithelial barrier function following *S. pneumoniae* infection (110) Chronic type III IFN stimulation of lung epithelial cells leads to loss of barrier function and bacteria infiltration (115, 116)	Type I and III IFNs help to maintain the intestinal epithelial barrier function following *Salmonella enterica* serovar *Typhimurium* infection. Type III IFN is more potent in promoting barrier function (111)
Microbiota	Currently unknown if bacteria play a role in shaping interferon responses in the lung	Microbiota promote norovirus persistent infection via modulation of type III IFN signaling (100)

Seven type I IFNs (recombinant and pegylated IFN-α2a, IFN-α2b, IFN-β1a and IFN-β1b) have passed clinical trials and have been approved for use in treating virus infections (HBV, HCV), multiple sclerosis, leukemia, melanoma, and multiple myeloma (112). Currently three clinical trials are ongoing for type III IFNs, and while they are promising and have reported reduced side effects, they have still not been clinically approved for use in patients. IFN-α was key to early HCV treatment and its activity was improved by combining it with ribavirin and through pegylation (113). However, problems with non-responsive patients, drug toxicity and liver cells becoming refractive to IFN-α treatment has led it to become a second choice for HCV treatments (112). IFN-α has also been used to treat HBV where it has been shown to decrease viral loads in the blood and improve liver enzymes (114). While the pegylated form has also shown higher activity against HBV, patients experience similar side effects and loss of function as HCV patients. Recently, IFN-λ has been used in clinical trials against HCV, HBV and HDV. It has shown similar effects as IFN-α treatment in reducing viral loads, but patients describe less side effects. Longer term studies will be required to determine if patients also become refractory to IFN-λ treatment, however *in vitro* experiments suggest that IFN-λ does not lead to a loss of function even after cell cultures have had prolonged treatments which could be explained by the lack of negative regulators affecting type III IFN signaling (*e.g.* USP18) (79). Currently pegylated IFN-λ is in phase II clinical trials to assess its action against SARS-CoV-2. Many researchers are optimistic about its potential to act against SARS-CoV-2 as both IFNs have been shown to reduce replication however IFN-λ clears infection with less tissue damage that type I IFNs. However, we need to be careful as IFN-λ is not without risk as recent studies have highlighted that treatment with IFN-λ could prevent lung epithelial cell regeneration and favors bacterial superinfection (115, 116).

With age, mice do not rely on the type I IFN but rather the type III IFN to protect their intestinal epithelium against enteric pathogens (99). Additionally, when primary epithelial cells are isolated from the intestinal tract and cultured *in vitro* they regain responsiveness to type I IFNs (24). These observations suggest that the gut microenvironment (tissue specific immune cells, microbiota, hypoxia and peristalsis) is participating in regulating the IFN response in a precise manner. It was shown that during enteric virus infection of mice, innate lymphoid cells in the gut secrete IL-22 which can act on IECs to synergize the antiviral activity of type III IFN (97). It was shown that IL-22 enhances the expression of IFN-λ induced ISGs and this likely participates in amplifying the antiviral response (97). However, it is known that IL-22 is mostly a key cytokine for regulating cell proliferation and barrier function in the intestine (117). With the importance of type III IFN in regulating barrier function, it is now important to address whether the benefit of IL-22 is exerted *via* ISGs or due to improved tissue repair. Similarly, if IL-22 is also acting with IFN-λ in humans remains to be determined.

Hypoxia is a critical parameter of the gut which is often overlooked in infectious disease research. Hypoxia is not only required for the microbiota but it also influences the epithelial cells themselves (118). It was shown that hypoxia favors barrier function in human intestinal epithelial cells (119). As it is well established that hypoxia in the tumor microenvironment affects the response of immune cells (118), it is critical to start investigating whether hypoxia could impact immune response of intestinal epithelial cells upon infection.

An additional epithelium specific parameter that has been neglected up to now is the fact that epithelium surfaces are composed of multiple cell types. With the advance of both single cell sequencing technologies and organoid cultures, it is now possible to address whether different cell types lining epithelium surfaces mount a similar or distinct immune response upon pathogen challenges. Similarly, it will be important to investigate whether these different cell types will generate the same ISGs upon IFN stimulation to address if each individual cell type establishes a distinct antiviral strategy to preserve its cell type specific function. While there are single cell studies of viral infection in cell lines (120), understanding viral infection at the single cell level in the tissue or in organoids is in its infancy.

Finally, one of the most underappreciated parameters influencing IFN signaling is the stochastic response of cells following IFN stimulation and the heterogeneity in the generated response. Understanding this complex relationship between cell populations and IFN response requires not only biological approaches where the importance of different transcription factors will be addressed *via* genetic manipulation but also through the use of mathematical modeling to gain a system understanding of IFN signaling. This heterogeneity in response to IFN is even more complicated as the spatial location of an individual cell within a population seems to impact its response to IFN. It was shown that when murine IECs become confluent and polarized, they become more responsive to IFN-λ (92). This work should constitute a building block for future research directions as it is likely to have critical implications at mucosal surfaces as epithelium cells form a condensed polarized monolayer of cells. We can speculate, for example in the gut, depending on the intestinal section that you are looking at, depending on whether a cell is located in the crypt or villi region or if the tissue is damaged and there are microlesions, that there will be differences in how IECs respond to pathogens and secreted IFN.

## Author’s Note

The figures in this review were created using Biorender.

## Author Contributions

All authors contributed to the article and approved the submitted version.

## Funding

This work was supported by research grants from the Deutsche Forschungsgemeinschaft (DFG): project numbers 415089553 (Heisenberg program), 240245660 (SFB1129), 278001972 (TRR186), and 272983813 (TRR179) to SB; 416072091 to MS. We also acknowledge funding from the German Academic Exchange Service (DAAD) (Research Grant 57440921) to PD and the China Scholarship Council to CG.

## Conflict of Interest

The authors declare that the research was conducted in the absence of any commercial or financial relationships that could be construed as a potential conflict of interest.

## References

[1] Isaacs A Lindenmann J Virus interference I. interferon. Proc R Soc Lond B Biol Sci (1957) 147:258–67. 10.1098/rspb.1957.0048 26297790

[2] Schoggins JW Rice CM Interferon-stimulated genes and their antiviral effector functions. Curr Opin Virol (2011) 1:519–25. 10.1016/j.coviro.2011.10.008 PMC3274382 22328912

[3] Schoggins JW Interferon-Stimulated Genes: What Do They All Do? Annu Rev Virol (2019) 6:567–84. 10.1146/annurev-virology-092818-015756 31283436

[4] Isaacs A Lindenmann J Virus interference. I. The interferon. By A. Isaacs and J. Lindenmann. J Interferon Res (1987) 7:429–38. 10.1089/jir.1987.7.429 2445832

[5] LaFleur DW Nardelli B Tsareva T Mather D Feng P Semenuk M Interferon-kappa novel type I interferon expressed human keratinocytes. J Biol Chem (2001) 276:39765–71. 10.1074/jbc.M102502200 11514542

[6] Takaoka A Yanai H Interferon signalling network in innate defence. Cell Microbiol (2006) 8:907–22. 10.1111/j.1462-5822.2006.00716.x 16681834

[7] Hoffmann H-H Schneider WM Rice CM Interferons and viruses: an evolutionary arms race of molecular interactions. Trends Immunol (2015) 36:124–38. 10.1016/j.it.2015.01.004 PMC4384471 25704559

[8] Kyogoku C Smiljanovic B Grün JR Biesen R Schulte-Wrede U Häupl T Cell-specific type I IFN signatures in autoimmunity and viral infection: what makes the difference? PloS One (2013) 8:e83776. 10.1371/journal.pone.0083776 24391825 PMC3877094

[9] Gibbert K Schlaak JF Yang D Dittmer U IFN-α subtypes: distinct biological activities in anti-viral therapy. Br J Pharmacol (2013) 168:1048–58. 10.1111/bph.12010 PMC3594665 23072338

[10] Novick D Cohen B Rubinstein M The human interferon alpha/beta receptor: characterization and molecular cloning. Cell. (1994) 77:391–400. 10.1016/0092-8674(94)90154-6 8181059

[11] Jaks E Gavutis M Uzé G Martal J Piehler J Differential receptor subunit affinities of type I interferons govern differential signal activation. J Mol Biol (2007) 366:525–39. 10.1016/j.jmb.2006.11.053 17174979

[12] de Weerd NA Nguyen T The interferons and their receptors–distribution and regulation. Immunol Cell Biol (2012) 90:483–91. 10.1038/icb.2012.9 PMC7165917 22410872

[13] Langer V Vivi E Regensburger D Winkler TH Waldner MJ Rath T IFN-γ drives inflammatory bowel disease pathogenesis through VE-cadherin-directed vascular barrier disruption. J Clin Invest (2019) 129:4691–707. 10.1172/JCI124884 PMC6819119 31566580

[14] Pestka S Kotenko SV Muthukumaran G Izotova LS Cook JR Garotta G The interferon gamma (IFN-gamma) receptor: a paradigm for the multichain cytokine receptor. Cytokine Growth Factor Rev (1997) 8:189–206. 10.1016/S1359-6101(97)00009-9 9462485

[15] Alspach E Lussier DM Schreiber RD Interferon γ and Its Important Roles in Promoting and Inhibiting Spontaneous and Therapeutic Cancer Immunity. Cold Spring Harb Perspect Biol (2019) 11:1–20. 10.1101/cshperspect.a028480 PMC6396335 29661791

[16] Kotenko SV Gallagher G Baurin VV Lewis-Antes A Shen M Shah NK IFN-lambdas mediate antiviral protection through a distinct class II cytokine receptor complex. Nat Immunol (2003) 4:69–77. 10.1038/ni875 12483210

[17] Sheppard P Kindsvogel W Xu W Henderson K Schlutsmeyer S Whitmore TE IL-28, IL-29 and their class II cytokine receptor IL-28R. Nat Immunol (2003) 4:63–8. 10.1038/ni873 12469119

[18] Prokunina-Olsson L Muchmore B Tang W Pfeiffer RM Park H Dickensheets H A variant upstream IFNL3 (IL28B) creating new interferon gene IFNL4 is associated with impaired clearance of hepatitis C virus. Nat Genet (2013) 45:164–71. 10.1038/ng.2521 PMC3793390 23291588

[19] Onabajo OO Muchmore B Prokunina-Olsson L The IFN-λ4 Conundrum: When a Good Interferon Goes Bad. J Interferon Cytokine Res (2019) 39:636–41. 10.1089/jir.2019.0044 PMC6767864 31241411

[20] Ansari MA Pedergnana V Ip CLC Magri A Von Delft A Bonsall D Genome-to-genome analysis highlights the effect of the human innate and adaptive immune systems on the hepatitis C virus Nat Genet (2017) 49:666–73. 10.1038/ng.3835 PMC5873514 28394351

[21] Ansari MA Aranday-Cortes E Ip CL da Silva Filipe A Lau SH Bamford C Interferon lambda 4 impacts the genetic diversity of hepatitis C virus. eLife (2019) 8:e42463. 10.7554/eLife.42463 31478835 PMC6721795

[22] Bamford CGG Aranday-Cortes E Filipe IC Sukumar S Mair D Filipe A da S A polymorphic residue that attenuates the antiviral potential of interferon lambda 4 in hominid lineages. PloS Pathog (2018) 14:e1007307. 10.1371/journal.ppat.1007307 30308076 PMC6181419

[23] Sommereyns C Paul S Staeheli P Michiels T IFN-lambda (IFN-lambda) is expressed in a tissue-dependent fashion and primarily acts on epithelial cells in vivo. PloS Pathog (2008) 4:e1000017. 10.1371/journal.ppat.1000017 18369468 PMC2265414

[24] Pott J Mahlakõiv T Mordstein M Duerr CU Michiels T Stockinger S IFN-lambda determines the intestinal epithelial antiviral host defense. Proc Natl Acad Sci USA (2011) 108:7944–9. 10.1073/pnas.1100552108 PMC3093475 21518880

[25] Mordstein M Neugebauer E Ditt V Jessen B Rieger T Falcone V Lambda interferon renders epithelial cells of the respiratory and gastrointestinal tracts resistant to viral infections. J Virol (2010) 84:5670–7. 10.1128/JVI.00272-10 PMC2876583 20335250

[26] Zhou Z Hamming OJ Ank N Paludan SR Nielsen AL Hartmann R Type III interferon (IFN) induces a type I IFN-like response in a restricted subset of cells through signaling pathways involving both the Jak-STAT pathway and the mitogen-activated protein kinases. J Virol (2007) 81:7749–58. 10.1128/JVI.02438-06 PMC1933366 17507495

[27] Koltsida O Hausding M Stavropoulos A Koch S Tzelepis G Ubel C (IFN-λ2) modulates lung DC function to promote Th1 immune skewing and suppress allergic airway disease. EMBO Mol Med (2011) 3:348–61. 10.1002/emmm.201100142 PMC3377081 21538995

[28] Blazek K Eames HL Weiss M Byrne AJ Perocheau D Pease JE IFN-λ resolves inflammation via suppression of neutrophil infiltration and IL-1β production. J Exp Med (2015) 212:845–53. 10.1084/jem.20140995 PMC4451128 25941255

[29] Galani IE Triantafyllia V Eleminiadou E-E Koltsida O Stavropoulos A Manioudaki M Interferon-λ Mediates Non-redundant Front-Line Antiviral Protection against Influenza Virus Infection without Compromising Host Fitness. Immunity (2017) 46:875–90.e6. 10.1016/j.immuni.2017.04.025 28514692

[30] Broggi A Tan Y Granucci F Zanoni I IFN-λ suppresses intestinal inflammation by non-translational regulation of neutrophil function. Nat Immunol (2017) 18:1084–93. 10.1038/ni.3821 PMC5701513 28846084

[31] Santer DM Minty GES Golec DP Lu J May J Namdar A Differential expression of interferon-lambda receptor 1 splice variants determines the magnitude of the antiviral response induced by interferon-lambda 3 in human immune cells. PloS Pathog (2020) 16:e1008515. 10.1371/journal.ppat.1008515 32353085 PMC7217487

[32] Chow KT Gale M Jr Loo Y-M RIG-I and Other RNA Sensors in Antiviral Immunity. Annu Rev Immunol (2018) 36:667–94. 10.1146/annurev-immunol-042617-053309 29677479

[33] Levy DE Marié IJ Durbin JE Induction and function of type I and III interferon in response to viral infection. Curr Opin Virol (2011) 1:476–86. 10.1016/j.coviro.2011.11.001 PMC3272644 22323926

[34] Rehwinkel J Gack MU RIG-I-like receptors: their regulation and roles in RNA sensing. Nat Rev Immunol (2020) 20:537–51. 10.1038/s41577-020-0288-3 PMC7094958 32203325

[35] Dolasia K Bisht MK Pradhan G Udgata A Mukhopadhyay S TLRs/NLRs: Shaping the landscape of host immunity. Int Rev Immunol (2018) 37:3–19. 10.1080/08830185.2017.1397656 29193992

[36] Boulant S Stanifer M Lozach P-Y Dynamics of virus-receptor interactions in virus binding, signaling, and endocytosis. Viruses (2015) 7:2794–815. 10.3390/v7062747 PMC4488714 26043381

[37] Kagan JC Su T Horng T Chow A Akira S Medzhitov R TRAM couples endocytosis of Toll-like receptor 4 to the induction of interferon-beta. Nat Immunol (2008) 9:361–8. 10.1038/ni1569 PMC4112825 18297073

[38] Dixit E Boulant S Zhang Y Lee ASY Odendall C Shum B Peroxisomes are signaling platforms for antiviral innate immunity. Cell (2010) 141:668–81. 10.1016/j.cell.2010.04.018 PMC3670185 20451243

[39] Odendall C Dixit E Stavru F Bierne H Franz KM Durbin AF Diverse intracellular pathogens activate type III interferon expression from peroxisomes Nat Immunol (2014) 15:717–26. 10.1038/ni.2915 PMC4106986 24952503

[40] Bender S Reuter A Eberle F Einhorn E Binder M Bartenschlager R Activation of Type I and III Interferon Response by Mitochondrial and Peroxisomal MAVS and Inhibition by Hepatitis C Virus. PloS Pathog (2015) 11:e1005264. 10.1371/journal.ppat.1005264 26588843 PMC4654527

[41] Stanifer ML Mukenhirn M Muenchau S Pervolaraki K Kanaya T Albrecht D Asymmetric distribution of TLR3 leads to a polarized immune response in human intestinal epithelial cells. Nat Microbiol (2020) 5:181–91. 10.1038/s41564-019-0594-3 31686029

[42] O’Neal JT Upadhyay AA Wolabaugh A Patel NB Bosinger SE Suthar MS West Nile Virus-Inclusive Single-Cell RNA Sequencing Reveals Heterogeneity in the Type I Interferon Response within Single Cells. J Virol (2019) 93:e01778–18. 10.1128/JVI.01778-18 PMC6401468 30626670

[43] Zhao M Zhang J Phatnani H Scheu S Maniatis T Stochastic expression of the interferon-β gene. PloS Biol (2012) 10:e1001249. 10.1371/journal.pbio.1001249 22291574 PMC3265471

[44] Rand U Rinas M Schwerk J Nöhren G Linnes M Kröger A Multi-layered stochasticity and paracrine signal propagation shape the type-I interferon response. Mol Syst Biol (2012) 8:584. 10.1038/msb.2012.17 22617958 PMC3377992

[45] Schmid B Rinas M Ruggieri A Acosta EG Bartenschlager M Reuter A Live Cell Analysis and Mathematical Modeling Identify Determinants of Attenuation of Dengue Virus 2’-O-Methylation Mutant. PloS Pathog (2015) 11:e1005345. 10.1371/journal.ppat.1005345 26720415 PMC4697809

[46] Pervolaraki K Rastgou Talemi S Albrecht D Bormann F Bamford C Mendoza JL Differential induction of interferon stimulated genes between type I and type III interferons is independent of interferon receptor abundance. PloS Pathog (2018) 14:e1007420. 10.1371/journal.ppat.1007420 30485383 PMC6287881

[47] Jilg N Lin W Hong J Schaefer EA Wolski D Meixong J Kinetic differences in the induction of interferon stimulated genes by interferon-α and interleukin 28B are altered by infection with hepatitis C virus. Hepatology (2014) 59:1250–61. 10.1002/hep.26653 PMC3909557 23913866

[48] Bolen CR Ding S Robek MD Kleinstein SH Dynamic expression profiling of type I and type III interferon-stimulated hepatocytes reveals a stable hierarchy of gene expression. Hepatology (2014) 59:1262–72. 10.1002/hep.26657 PMC3938553 23929627

[49] Lavoie TB Kalie E Crisafulli-Cabatu S Abramovich R DiGioia G Moolchan K Binding activity all human alpha interferon subtypes. Cytokine (2011) 56:282–9. 10.1016/j.cyto.2011.07.019 21856167

[50] Piehler J Schreiber G Mutational and structural analysis of the binding interface between type I interferons and their receptor Ifnar2. J Mol Biol (1999) 294:223–37. 10.1006/jmbi.1999.3230 10556041

[51] Mendoza JL Schneider WM Hoffmann H-H Vercauteren K Jude KM Xiong A The IFN-λ-IFN-λR1-IL-10Rβ Complex Reveals Structural Features Underlying Type III IFN Functional Plasticity. Immun (2017) 46:379–92. 10.1016/j.immuni.2017.02.017 PMC5510750 28329704

[52] Yan H Krishnan K Lim JT Contillo LG Krolewski JJ Molecular characterization of an alpha interferon receptor 1 subunit (IFNaR1) domain required for TYK2 binding and signal transduction. Mol Cell Biol (1996) 16:2074–82. 10.1128/MCB.16.5.2074 PMC231194 8628273

[53] Barbieri G Velazquez L Scrobogna M Fellous M Pellegrini S Activation of the protein tyrosine kinase tyk2 by interferon alpha/beta. Eur J Biochem (1994) 223:427–35. 10.1111/j.1432-1033.1994.tb19010.x 8055912

[54] Wallweber HJA Tam C Franke Y Starovasnik MA Lupardus PJ Structural basis of recognition of interferon-α receptor by tyrosine kinase 2. Nat Struct Mol Biol (2014) 21:443–8. 10.1038/nsmb.2807 PMC4161281 24704786

[55] Finbloom DS Winestock KD IL-10 induces the tyrosine phosphorylation of tyk2 and Jak1 and the differential assembly of STAT1 alpha and STAT3 complexes in human T cells and monocytes. J Immunol (1995) 155:1079–90. 7543512

[56] Ho AS Wei SH Mui AL Miyajima A Moore KW Functional regions of the mouse interleukin-10 receptor cytoplasmic domain. Mol Cell Biol (1995) 15:5043–53. 10.1128/mcb.15.9.5043 PMC230751 7544437

[57] Domanski P Fish E Nadeau OW Witte M Platanias LC Yan H A region of the beta subunit of the interferon alpha receptor different from box 1 interacts with Jak1 and is sufficient to activate the Jak-Stat pathway and induce an antiviral state. J Biol Chem (1997) 272:26388–93. 10.1074/jbc.272.42.26388 9334213

[58] Ferrao R Wallweber HJA Ho H Tam C Franke Y Quinn J The Structural Basis for Class II Cytokine Receptor Recognition by JAK1. Structure (2016) 24:897–905. 10.1016/j.str.2016.03.023 27133025 PMC4899269

[59] Rodig SJ Meraz MA White JM Lampe PA Riley JK Arthur CD Disruption of the Jak1 gene demonstrates obligatory and nonredundant roles of the Jaks in cytokine-induced biologic responses. Cell (1998) 93:373–83. 10.1016/S0092-8674(00)81166-6 9590172

[60] Eletto D Burns SO Angulo I Plagnol V Gilmour KC Henriquez F Biallelic JAK1 mutations in immunodeficient patient with mycobacterial infection. Nat Commun (2016) 7:13992. 10.1038/ncomms13992 28008925 PMC5196432

[61] Kreins AY Ciancanelli MJ Okada S Kong X-F Ramírez-Alejo N Kilic SS Human TYK2 deficiency: Mycobacterial and viral infections without hyper-IgE syndrome. J Exp Med (2015) 212:1641–62. 10.1084/jem.20140280 PMC4577846 26304966

[62] Karaghiosoff M Neubauer H Lassnig C Kovarik P Schindler H Pircher H Partial impairment of cytokine responses in Tyk2-deficient mice. Immunity (2000) 13:549–60. 10.1016/S1074-7613(00)00054-6 11070173

[63] Fuchs S Kaiser-Labusch P Bank J Ammann S Kolb-Kokocinski A Edelbusch C Tyrosine kinase 2 is not limiting human antiviral type III interferon responses. Eur J Immunol (2016) 46:2639–49. 10.1002/eji.201646519 27615517

[64] Watling D Guschin D Müller M Silvennoinen O Witthuhn BA Quelle FW Complementation by the protein tyrosine kinase JAK2 of a mutant cell line defective in the interferon-gamma signal transduction pathway. Nature (1993) 366:166–70. 10.1038/366166a0 7901766

[65] Darnell JE Jr Kerr IM Stark GR Jak-STAT pathways and transcriptional activation in response to IFNs and other extracellular signaling proteins. Science (1994) 264:1415–21. 10.1126/science.8197455 8197455

[66] Darnell JE Jr STATs and gene regulation. Sci (1997) 277:1630–5. 10.1126/science.277.5332.1630 9287210

[67] Meinke A Barahmand-Pour F Wöhrl S Stoiber D Decker T Activation of different Stat5 isoforms contributes to cell-type-restricted signaling in response to interferons. Mol Cell Biol (1996) 16:6937–44. 10.1128/MCB.16.12.6937 PMC231697 8943349

[68] Fasler-Kan E Pansky A Wiederkehr M Battegay M Heim MH Interferon-alpha activates signal transducers and activators of transcription 5 and 6 in Daudi cells. Eur J Biochem (1998) 254:514–9. 10.1046/j.1432-1327.1998.2540514.x 9688261

[69] Farrar JD Smith JD Murphy TL Murphy KM Recruitment of Stat4 to the Human Interferon-α/β Receptor Requires Activated Stat2. J Biol Chem (2000) 275:2693–7. 10.1074/jbc.275.4.2693 10644731

[70] Kotenko SV IFN-λs. Curr Opin Immunol (2011) 23:583–90. 10.1016/j.coi.2011.07.007 PMC3196341 21840693

[71] Pervolaraki K Stanifer ML Münchau S Renn LA Albrecht D Kurzhals S Type I and Type III Interferons Display Different Dependency on Mitogen-Activated Protein Kinases to Mount an Antiviral State in the Human Gut. Front Immunol (2017) 8:459. 10.3389/fimmu.2017.00459 28484457 PMC5399069

[72] Sanchez GAM Reinhardt A Ramsey S Wittkowski H Hashkes PJ Berkun Y JAK1/2 inhibition with baricitinib in the treatment of autoinflammatory interferonopathies. J Clin Invest (2018) 128:3041–52. 10.1172/JCI98814 PMC6026004 29649002

[73] Papp K Gordon K Thaçi D Morita A Gooderham M Foley P Phase 2 Trial of Selective Tyrosine Kinase 2 Inhibition in Psoriasis. N Engl J Med (2018)379:1313–21. 10.1056/NEJMoa1806382 30205746

[74] Fenner JE Starr R Cornish AL Zhang J-G Metcalf D Schreiber RD Suppressor of cytokine signaling 1 regulates the immune response toinfection by a unique inhibition of type I interferon activity. NatImmunol (2006) 7:33–9. 10.1038/ni1287 16311601

[75] Piganis RAR De Weerd NA Gould JA Schindler CW Mansell A Nicholson SE Suppressor of cytokine signaling (SOCS) 1 inhibits type I interferon(IFN) signaling via the interferon alpha receptor (IFNAR1)-associated tyrosine kinase Tyk2. J Biol Chem (2011) 286:33811–8. 10.1074/jbc.M111.270207 PMC3190811 21757742

[76] Blumer T Coto-Llerena M Duong FHT Heim MH SOCS1 is an inducible negative regulator of interferon λ (IFN-λ)-induced gene expression in vivo. J Biol Chem (2017) 292:17928–38. 10.1074/jbc.M117.788877 PMC5663890 28900038

[77] François-Newton V Magno de Freitas Almeida G Payelle-Brogard B Monneron D Pichard-Garcia L Piehler J USP18-based negative feedback control is induced by type I and type III interferons and specifically inactivates interferon α response. PloS One (2011) 6:e22200. 10.1371/journal.pone.0022200 21779393 PMC3136508

[78] Schreiber G Piehler J The molecular basis for functional plasticity in type I interferon signaling. Trends Immunol (2015) 36:139–49. 10.1016/j.it.2015.01.002 25687684

[79] Sarasin-Filipowicz M Wang X Yan M Duong FHT Poli V Hilton DJ Alpha interferon induces long-lasting refractoriness of JAK-STAT signaling in the mouse liver through induction of USP18/UBP43. Mol Cell Biol (2009) 29:4841–51. 10.1128/MCB.00224-09 PMC2725724 19564419

[80] Makowska Z Duong FHT Trincucci G Tough DF Heim MH Interferon-β and interferon-λ signaling is not affectedby interferon-induced refractoriness to interferon-α in vivo. Hepatology (2011) 53:1154–63. 10.1002/hep.24189 21480323

[81] Basters A Knobeloch K-P Fritz G USP18 - a multifunctional component in the interferon response. Biosci Rep (2018) 38:1–9. 10.1042/BSR20180250 PMC6240716 30126853

[82] Pervolaraki K Guo C Albrecht D Boulant S Stanifer ML Type-Specific Crosstalk Modulates Interferon Signaling in IntestinalEpithelial Cells. J Interferon Cytokine Res(2019) 39:650–60. 10.1089/jir.2019.0040 31199715

[83] Marcello T Grakoui A Barba-Spaeth G Machlin ES Kotenko SV MacDonald MR Interferons alpha and lambda inhibit hepatitis C virus replicationwith distinct signal transduction and gene regulation kinetics. Gastroenterology (2006) 131:1887–98. 10.1053/j.gastro.2006.09.052 17087946

[84] Selvakumar TA Bhushal S Kalinke U Wirth D Hauser H Köster M Identification of a Predominantly Interferon-λ-InducedTranscriptional Profile in Murine Intestinal Epithelial Cells. FrontImmunol (2017) 8:1302. 10.3389/fimmu.2017.01302 PMC5650613 29085367

[85] Forero A Ozarkar S Li H Lee CH Hemann EA Nadjsombati MS Differential Activation of the Transcription Factor IRF1 Underliesthe Distinct Immune Responses Elicited by Type I and Type III Interferons. Immunity (2019) 51:451–64.e6. 10.1016/j.immuni.2019.07.007 31471108 PMC7447158

[86] Novatt H Renn L Theisen T Massie T Massie T Rabin RL Interferon regulatory factor 1 (IRF1) expression patterns by respiratory epithelial cells reveal non-redundancy of type I versus type III interferon. J Immunol (2016) 196:68.12–2. 10.3390/ijms20061445

[87] Novatt H Theisen TC Massie T Massie T Simonyan V Voskanian-Kordi A Distinct Patterns of Expression Transcription Factors in Response to Interferonβ and Interferonλ1. J Interferon Cytokine Res (2016) 36:589–98. 10.1089/jir.2016.0031 27447339

[88] Raj A Peskin CS Tranchina D Vargas DY Tyagi S Stochastic mRNA synthesis in mammalian cells. PloS Biol (2006) 4:e309. 10.1371/journal.pbio.0040309 17048983 PMC1563489

[89] Mariani L Schulz EG Lexberg MH Helmstetter C Radbruch A Löhning M Short-term memory in gene induction reveals the regulatory principle behind stochastic IL-4 expression. Mol Syst Biol (2010) 6:359. 10.1038/msb.2010.13 20393579 PMC2872609

[90] Bauhofer O Ruggieri A Schmid B Schirmacher P Bartenschlager R Persistence of HCV in quiescent hepatic cells under conditions of aninterferon-induced antiviral response. Gastroenterology (2012) 143:429–38.e8. 10.1053/j.gastro.2012.04.018 22522091

[91] Maiwald T Schneider A Busch H Sahle S Gretz N Weiss TS Combining theoretical analysis and experimental data generationreveals IRF9 as a crucial factor for accelerating interferon α-induced early antiviralsignalling. FEBS J (2010) 277:4741–54. 10.1111/j.1742-4658.2010.07880.x 20964804

[92] Bhushal S Wolfsmüller M Selvakumar TA Kemper L Wirth D Hornef MW Cell Polarization and Epigenetic Status Shape the Heterogeneous Response to Type III Interferons in Intestinal Epithelial Cells. FrontImmunol (2017) 8:671. 10.3389/fimmu.2017.00671 PMC5467006 28659914

[93] Lazear HM Schoggins JW Diamond MS Shared and Distinct Functions of Type I and Type III Interferons. Immunity (2019) 50:907–23. 10.1016/j.immuni.2019.03.025 PMC6839410 30995506

[94] Nice TJ Baldridge MT McCune BT Norman JM Lazear HM Artyomov M Interferon-λ cures persistent murine norovirus infection inthe absence of adaptive immunity. Science (2015) 347:269–73. 10.1126/science.1258100 PMC4398891 25431489

[95] Mahlakõiv T Hernandez P Gronke K Diefenbach A Staeheli P Leukocyte-derived IFN-α/β and epithelial IFN-λconstitute a compartmentalized mucosal defense system that restricts enteric virusinfections. PloS Pathog (2015) 11:e1004782. 10.1371/journal.ppat.1004782 25849543 PMC4388470

[96] Stanifer ML Kee C Cortese M Zumaran CM Triana S Mukenhirn M Critical Role of Type III Interferon in Controlling SARS-CoV-2 Infection in Human Intestinal Epithelial Cells. Cell Rep (2020) 32:107863. 10.1016/j.celrep.2020.107863 32610043 PMC7303637

[97] Hernández PP Mahlakoiv T Yang I Schwierzeck V Nguyen N Guendel F Interferon-λ and interleukin 22 act synergistically for theinduction of interferon-stimulated genes and control of rotavirus infection. Nat Immunol (2015) 16:698–707. 10.1038/ni.3180 26006013 PMC4589158

[98] Baldridge MT Lee S Brown JJ McAllister N Urbanek K Dermody TS Expression of Ifnlr1 on Intestinal Epithelial Cells Is Critical to the Antiviral Effects of Interferon Lambda against Norovirus and Reovirus. J Virol (2017) 91:e02079–16. 10.1128/JVI.02079-16 PMC5355594 28077655

[99] Lin J-D Feng N Sen A Balan M Tseng H-C McElrath C Distinct Roles of Type I and Type III Interferons in IntestinalImmunity to Homologous and Heterologous Rotavirus Infections. PloS Pathog (2016) 12:e1005600. 10.1371/journal.ppat.1005600 27128797 PMC4851417

[100] Baldridge MT Nice TJ McCune BT Yokoyama CC Kambal A Wheadon M Commensal microbes and interferon-λ determine persistence ofenteric murine norovirus infection. Science (2015)347:266–9. 10.1126/science.1258025 PMC4409937 25431490

[101] Tschurtschenthaler M Wang J Fricke C Fritz TMJ Niederreiter L Adolph TE Type I interferon signalling in the intestinal epithelium affectsPaneth cells, microbial ecology and epithelial regeneration. Gut (2014) 63:1921–31. 10.1136/gutjnl-2013-305863 24555997

[102] Saxena K Simon LM Zeng X-L Blutt SE Crawford SE Sastri NP A paradox of transcriptional and functional innate interferonresponses of human intestinal enteroids to enteric virus infection. Proc Natl Acad Sci USA (2017) 114:E570–9. 10.1073/pnas.1615422114 PMC5278484 28069942

[103] Hosmillo M Chaudhry Y Nayak K Sorgeloos F Koo B-K Merenda A Norovirus Replication in Human Intestinal Epithelial Cells Is Restricted by the Interferon-Induced JAK/STAT Signaling Pathway and RNA Polymerase II-Mediated Transcriptional Responses. MBio (2020) 11:e00215–20. 10.1128/mBio.00215-20 PMC7078467 32184238

[104] Crotta S Davidson S Mahlakoiv T Desmet CJ Buckwalter MR Albert ML Type I and type III interferons drive redundant amplification loops to induce a transcriptional signature in influenza-infected airway epithelia. PloS Pathog (2013) 9:e1003773. 10.1371/journal.ppat.1003773 24278020 PMC3836735

[105] Klinkhammer J Schnepf D Ye L Schwaderlapp M Gad HH Hartmann R IFN-λ prevents influenza virus spread from the upper airways to the lungs and limits virus transmission. Elife (2018) 7:e33354. 10.7554/eLife.33354 29651984 PMC5953542

[106] Ye L Schnepf D Becker J Ebert K Tanriver Y Bernasconi V Interferon-λ enhances adaptive mucosal immunity by boosting release of thymic stromal lymphopoietin. Nat Immunol (2019) 20:593–601. 10.1038/s41590-019-0345-x 30886417

[107] Ye L Ohnemus A Ong LC Gad HH Hartmann R Lycke N Type I and Type III Interferons Differ in Their Adjuvant Activities for Influenza Vaccines. J Virol (2019) 93:e01262–19. 10.1128/JVI.01262-19 PMC6854507 31511392

[108] Espinosa V Dutta O McElrath C Du P Chang Y-J Cicciarelli B Type III interferon is a critical regulator of innate antifungal immunity. Sci Immunol (2017) eaan5357. 10.1126/sciimmunol.aan5357 PMC5880030 28986419

[109] Ye L Schnepf D Staeheli P Interferon-λ orchestrates innate and adaptive mucosal immuneresponses. Nat Rev Immunol (2019) 19:614–25. 10.1038/s41577-019-0182-z 31201377

[110] LeMessurier KS Häcker H Chi L Tuomanen E Redecke V Type I interferon protects against pneumococcal invasive disease byinhibiting bacterial transmigration across the lung. PloS Pathog (2013) 9:e1003727. 10.1371/journal.ppat.1003727 24244159 PMC3820719

[111] Odendall C Voak AA Kagan JC Type III IFNs Are Commonly Induced by Bacteria-Sensing TLRs and Reinforce Epithelial Barriers during Infection. J Immunol (2017) 199:3270–9 10.4049/jimmunol.1700250 PMC5679450 28954888

[112] Sarasin-Filipowicz M Oakeley EJ Duong FHT Christen V Terracciano L Filipowicz W Interferon signaling and treatment outcome in chronic hepatitis C. Proc Natl Acad Sci USA (2008) 105:7034–9 10.1073/pnas.0707882105 PMC2383932 18467494

[113] McHutchison JG Lawitz EJ Shiffman ML Muir AJ Galler GW McCone J Peginterferon alfa-2b or alfa-2a with ribavirin for treatment ofhepatitis C infection. N Engl J Med (2009) 361:580–93 10.1056/NEJMoa0808010 19625712

[114] Cooksley WGE Piratvisuth T Lee S-D Mahachai V Chao Y-C Tanwandee T Peginterferon alpha-2a (40 kDa): an advance in the treatment ofhepatitis B e antigen-positive chronic hepatitis B. J Viral Hepat (2003) 10:298–305. 10.1046/j.1365-2893.2003.00450.x 12823597

[115] Major J Crotta S Llorian M McCabe TM Gad HH Priestnall SL Type I and III interferons disrupt lung epithelial repair duringrecovery from viral infection. Science (2020) 369:712–7. 10.1126/science.abc2061 PMC7292500 32527928

[116] Broggi A Ghosh S Sposito B Spreafico R Balzarini F Lo Cascio A Type III interferons disrupt the lung epithelial barrier upon viral recognition. Science (2020) 369:706–12. 10.1126/science.abc3545 PMC7292499 32527925

[117] Lindemans CA Calafiore M Mertelsmann AM O’Connor MH Dudakov JA Jenq RR Interleukin-22 promotes intestinal-stem-cell-mediated epithelial regeneration. Nature (2015) 528:560–4. 10.1038/nature16460 PMC4720437 26649819

[118] Taylor CT Colgan SP Regulation of immunity and inflammation by hypoxia in immunologicalniches. Nat Rev Immunol (2017) 17:774–85. 10.1038/nri.2017.103 PMC5799081 28972206

[119] Muenchau S Deutsch R de Castro IJ Hielscher T Heber N Niesler B Hypoxic Environment Promotes Barrier Formation in Human Intestinal Epithelial Cells through Regulation of MicroRNA 320a Expression. Mol Cell Biol (2019) 39:e00553–18. 10.1128/MCB.00553-18 PMC6597885 31061092

[120] Wyler E Franke V Menegatti J Kocks C Boltengagen A Praktiknjo S Single-cell RNA-sequencing of herpes simplex virus 1-infected cellsconnects NRF2 activation to an antiviral program. Nat Commun (2019) 10:4878. 10.1038/s41467-019-12894-z 31653857 PMC6814756

